# Early discharge, inpatient treatment, or ICU management for patients with acute pulmonary embolism: is the guideline-recommended practice being followed?

**DOI:** 10.36416/1806-3756/e20240167

**Published:** 2025-05-05

**Authors:** Paula Ruiz-Talero, Oscar M Muñoz-Velandia, Santiago Méndez Salazar

**Affiliations:** 1. Departamento de Medicina Interna, Pontificia Universidad Javeriana, Bogotá, Colombia.; 2. Departamento de Medicina Interna, Hospital Universitario San Ignacio, Bogotá, Colombia.

**Keywords:** Pulmonary embolism, Practice guidelines as topic, Mortality, Patient care, Anticoagulants

## Abstract

**Objective:**

To describe the rates of adherence to the 2019 European Society of Cardiology guideline recommendations on the setting of care for patients with acute pulmonary embolism (PE) of varying severity.

**Methods:**

This was a retrospective cohort study of PE patients treated in a referral hospital in Colombia between 2019 and 2022.

**Results:**

We analyzed 506 patients with acute PE (median age, 67 years). Of those, 58% were women, and 33% had a history of cancer. In-hospital mortality was 9.2%, and 30-day mortality was 17.9%. Of the total of patients, 8.3% were classified as low-risk patients, 77.6% were classified as intermediate-low-risk patients, 11.2% were classified as intermediate-high-risk patients, and 2.7% were classified as high-risk patients. Of the total of low-risk patients, 9.5% were discharged early in accordance with the guideline recommendations. Of the total of intermediate-high-risk patients, 43.8% were treated in the general ward instead of being transferred to the ICU for monitoring. Of the total of high-risk patients, 92.8% were treated in the ICU. No cancer patients were discharged early.

**Conclusions:**

These results suggest that clinical practice guideline recommendations regarding the setting of care for patients with acute PE are not being followed. This is particularly true for low-risk PE patients who may be candidates for early discharge. Further studies are needed to investigate the reasons why low-risk patients are not being discharged early.

## INTRODUCTION

Venous thromboembolism, represented by pulmonary embolism (PE) and deep vein thrombosis, is the third most common cause of cardiovascular disease worldwide after acute myocardial infarction and stroke, affecting more than 300,000 people annually in the United States,^1^ ^,^ ^2^ with an all-cause hospital mortality of 14.8% in Colombia.^3^ This leads to high hospitalization rates and an approximate cost of $8,764 per patient, representing more than $2 billion in annual expenditures for its care in the United States between 2003 and 2010.^1^ Over the past 15 years, the mortality rate for PE has decreased as a result of advances in management strategies and early diagnosis, although the annual incidence continues to increase. In the general population, the incidence is estimated to be approximately 39-115 per 100,000 population per year, and it can be up to eight times higher in people > 80 years of age. 

There are several PE risk classification tools based on patient hemodynamic status and comorbidities. The Pulmonary Embolism Severity Index (PESI) and its simplified version (sPESI) are two of the most widely used scales predicting 30-day mortality. A combination of the clinical parameters assessed by the PESI/sPESI and the presence or absence of right ventricular dysfunction or positive biomarkers determines the final classification of patients as high-risk, intermediate-high-risk, intermediate-low-risk, or low-risk patients in accordance with the 2019 European Society of Cardiology (ESC) guidelines for the diagnosis and management of acute PE.^4^ The risk-adapted management strategies proposed in the guidelines include the setting of care, which may be inpatient treatment (in the general ward or ICU) or outpatient home treatment.^1^ ^,^ ^4^

Although a patient with PE can be correctly stratified on the basis of the PESI and sPESI, the current literature suggests that the recommendations of clinical practice guidelines regarding the setting of care for patients with acute PE are not always followed. For example, 80-90% of low-risk patients remain in hospital 24 h after admission, although 30-55% would be candidates for early discharge.^5^ ^,^ ^6^ In an observational study conducted in Italy between 2006 and 2013, the PESI did not significantly affect the rate or duration of hospitalization in low-risk patients.^7^ Given that there is a gap in knowledge regarding adherence to guideline recommendations for PE management in Latin America, the objective of the present study is to describe the rates of adherence to the 2019 ESC guideline recommendations on the setting of care for patients diagnosed with acute PE in a referral hospital in Colombia between 2019 and 2022. 

## METHODS

We performed a retrospective cohort study evaluating all patients with a diagnosis of acute PE admitted to the emergency department of the *Hospital Universitario San Ignacio*, in Bogotá, Colombia, between September of 2019 and July of 2022. Patients > 18 years of age with a diagnosis of acute PE based on CT pulmonary angiography or ventilation/perfusion scan findings were included. Patients with a diagnosis of acute PE related to SARS-CoV-2 infection were excluded because it has been documented that the PESI underestimates mortality in COVID-19 patients.^8^ Informed consent was waived for the present study, which was approved by the local research ethics committee (Protocol no. FM-CIE-0354-23). 

Patients were identified through the *Hospital Universitario San Ignacio* Anticoagulation Registry. This institutional registry includes all patients starting anticoagulant therapy for any diagnosis in the hospital. For the present study, patients with a diagnosis of acute PE were selected. Sociodemographic data and data on comorbidities, diagnostic test reports, anticoagulation use, and the sPESI were retrieved from the registry, where information is systematically collected at the point of care using standardized instruments. The *Hospital Universitario San Ignacio* Research Electronic Data Capture (REDCap; Vanderbilt University, Nashville, TN, USA) database and tools were used in order to collect and manage the data.^9^ ^,^ ^10^ Regular audits of the data collection process were performed to identify areas for improvement and ensure data quality. Missing information was supplemented with data from the institutional electronic medical records, which were retrospectively reviewed. 

The 2019 ESC guidelines for the diagnosis and management of acute PE were used for PE risk stratification,^4^ as follows. Low-risk patients were defined as having no hemodynamic instability at presentation, an sPESI of 0, no right ventricular dysfunction, and negative biomarkers. Intermediate-low-risk patients were defined as having no hemodynamic instability at presentation, an sPESI ≥ 1, no right ventricular dysfunction, and negative biomarkers (or positivity for only one of two parameters). Intermediate-high-risk patients were defined as having no hemodynamic instability at presentation, an sPESI ≥ 1, right ventricular dysfunction, and positive biomarkers. High-risk patients were defined as having hemodynamic instability at presentation or meeting any other criteria defined in the 2019 ESC guidelines.^4^ Right ventricular dysfunction was defined on the basis of the echocardiographic or imaging criteria in the 2019 ESC guidelines.^4^ Data on 30-day mortality were obtained from the Colombian *Administradora de los Recursos del Sistema General de Seguridad Social en Salud* (Social Security Administration) database, which systematically records the date of death of patients in the social security system in Colombia.^11^

Adherence to the 2019 ESC guideline recommendations on the setting of care for patients with acute PE of varying severity^4^ was evaluated. Adherence to the recommendations was considered high if low-risk patients were discharged within 24 h of admission; if intermediate-low-risk patients were admitted to the general ward; if intermediate-high-risk patients were closely monitored in the ICU; and if high-risk patients were monitored in the ICU. In addition, information on treatment, type of anticoagulation, and thrombolysis was collected. 

Absolute and relative frequencies were used in order to describe qualitative variables. Measures of central tendency and dispersion were calculated for quantitative variables (mean and standard deviation for variables with normal distribution and median and interquartile range for variables with non-normal distribution). The Shapiro-Wilk test was used in order to assess the normality assumption. Categorical variables are presented as absolute numbers and percentages. Adherence to the 2019 ESC guideline recommendations is presented as percentages and absolute numbers for each risk group. Statistical analysis was performed with Stata software, version 16 (StataCorp LLC, College Station, TX, USA). 

## RESULTS

A total of 506 patients were included in the present study. Their clinical and demographic characteristics are described in Table 1. All patients with acute PE diagnosed in our hospital and meeting the eligibility criteria were included. There were no missing data issues. The median age of the patients was 67 years, with hypertension being the most common comorbidity. Of the 506 patients included in the present study, 31.4% had concomitant deep vein thrombosis, 33.4% had a history of cancer, and the vast majority (85%) had active cancer at the time of diagnosis of PE. The most common cancer subtypes were gastrointestinal cancer (in 23.6%), genitourinary cancer (in 21.8%), breast cancer (in 12.4%), and hematologic cancer (in 10.6%). Most (77.6%) of the patients were classified as being intermediate-low-risk patients. 

**Table 1:** Sociodemographic and clinical characteristics of patients with pulmonary embolism.^a^

Variable	n = 506
Female sex	294 (58.1)
Age, years	67 [54-76]
Comorbidities	
Diabetes mellitus	61 (12.0)
Uncontrolled hypertension	192 (37.9)
Kidney failure	15 (2.9)
GFR (mL/min)^b^	
< 30	22 (4.4)
30-60	129 (26.2)
> 60	340 (69.2)
Drugs	
Aspirin	62 (12.2)
P2Y12 inhibitors	5 (0.9)
NSAIDs	22 (4.3)
Cancer	169 (33.4)
Active cancer^c^	145 (85.2)
Cancer subtype^c^	
Gastrointestinal	40 (23.6)
Genitourinary	37 (21.8)
Breast	21 (12.4)
Hematologic	18 (10.6)
Lung	16 (9.4)
Head and neck	14 (8.2)
Skin	13 (7.6)
Other^d^	9 (5.3)
Central nervous system	1 (0.5)
Previous VTE	7 (1.3)
Concomitant DVT	159 (31.4)
Proximal DVT^e^	75 (47.1)
Distal DVT^e^	84 (52.8)
In-hospital mortality	47 (9.3)
30-day mortality	91 (17.9)
sPESI classification	
0 points	43 (8.5)
1 point	122 (24.1)
2 points	239 (47.2)
3 points	93 (18.3)
4 points	9 (1.7)
ESC classification	
Low risk	42 (8.3)
Intermediate-low risk	393 (77.6)
Intermediate-high risk	57 (11.2)
High risk	14 (2.7)

GFR: glomerular filtration rate; NSAIDs: nonsteroidal anti-inflammatory drugs; VTE: venous thromboembolism; DVT: deep vein thrombosis; sPESI: simplified Pulmonary Embolism Severity Index; and ESC: European Society of Cardiology. ^a^Data expressed as n (%) or median [IQR]. ^b^Creatinine clearance measured with the Cockcroft-Gault formula. ^c^Among all cancer patients. ^d^Including sarcomas, neuroendocrine tumors, undifferentiated tumors, and metastatic disease with unknown primary. ^e^Among all deep vein thrombosis cases.

Table 2 shows clinical and paraclinical characteristics of interest, by PE severity. The frequency of anemia and thrombocytopenia increased with PE severity. All high-risk patients and 3.9% of the intermediate-high-risk patients met the 2019 ESC guideline definition of hemodynamic instability. Eighteen patients (4.5%) in the intermediate-low-risk group had hemodynamic instability for other causes, including septic shock (in 12) and hypovolemic shock (in 2). In addition, 57% of the high-risk patients received systemic thrombolysis. For the remaining patients, other reperfusion methods were used, such as thrombectomy and thrombolysis, because of the high risk of bleeding. All high-risk patients and some of the intermediate-high-risk patients were discussed with our PE response team. This led to two cases being reclassified because of echocardiographic evidence of chronic right ventricular dysfunction and because the hemodynamic instability was unlikely to be secondary to PE. None of the patients in other risk groups received reperfusion therapy. Biomarker positivity was not assessed in all high-risk patients, because they met the criteria for hemodynamic instability and right ventricular dysfunction. 

**Table 2:** Clinical and paraclinical characteristics of patients with pulmonary embolism of varying severity, as assessed by the European Society of Cardiology classification.

ESC classification	n	%	Cancer, n (%)*	Anemia^a^, n (%)*	Thrombocytopenia^b^, n (%)*	Positive troponin, n (%)*	Hemodynamic instability due to PE, n (%)*	Thrombolysis, n (%)*
Low risk	42	8.3	0 (0)	6 (14.2)	3 (7.1)	0 (0)	0 (0)	0 (0)
Intermediate- low risk	393	77.6	149 (37.9)	197 (50.1)	24 (6.1)	55 (13.9)	0 (0)	0 (0)
Intermediate-high risk	57	11.2	16 (28.0)	30 (52.6)	6 (10.5)	56 (98.2)	7 (3.9)	0 (0)
High risk	14	2.7	4 (28.5)	9 (64.2)	5 (35.7)	10 (71.4)	14 (100.0)	8 (57)
Total	506	100	169 (33.4)	242 (47.8)	38 (7.5)	121 (23.9)	21 (4.1)	8 (1.5)

ESC: European Society of Cardiology; and PE: pulmonary embolism. ^a^Defined as hemoglobin < 13 g/dL in men and < 12 g/dL in women. ^b^Defined as a platelet count < 150,000 per microliter. *The proportions refer to the total number of patients in each category.

In-hospital and 30-day mortality, length of hospital stay (in days), and bleeding complications increased with the risk of PE severity (Table 3). Notably, there was no 30-day mortality or bleeding complications in the low-risk group. Of those who died in hospital, 46% had a history of cancer, as did 57% of those who died at 30 days. 

**Table 3:** Outcomes in patients with pulmonary embolism of varying severity, as assessed by the European Society of Cardiology classification.

ESC classification	n	%	In-hospital mortality, n (%)*	30-day mortality, n (%)*	Hospital LOS, days Median [IQR]	Bleeding complications, n (%)*
Low risk	42	8.3	0 (0)	0 (0)	2 [2-7]	0 (0)
Intermediate-low risk	393	77.6	34 (8.6)	72 (18.3)	7 [4-13]	22 (5.6)
Intermediate-high risk	57	11.2	8 (14.0)	13 (22.8)	9 [6-15]	2 (3.5)
High risk	14	2.7	5 (35.7)	6 (42.8)	10.5 [3-15]	3 (21.4)
Total	506	100	47 (9.2)	91 (17.9)	7 [3-12]	27 (5.3)

ESC: European Society of Cardiology; and LOS: length of stay. *The proportions refer to the total number of patients in each category.

Adherence to the 2019 ESC guideline recommendations regarding the setting of care is shown in Table 4. Only 4 patients (9.5%) in the low-risk category were discharged early. The remaining patients were treated in the general ward. Only 56% of the intermediate-high-risk patients were monitored in the ICU, as were more than 90% of the high-risk patients. It is of note that 11.9% of the intermediate-low-risk patients were monitored in the ICU, most of them for reasons other than PE. 

**Table 4:** Adherence to the European Society of Cardiology-recommended setting of care for patients with acute pulmonary embolism of varying severity.

ESC classification	n	%	Setting of care		
			Early discharge, n (%)*	Hospitalization in the general ward, n (%)*	ICU, n (%)*
Low risk	42	8.3	4 (9.5)	37 (88.1)	1 (2.3)
Intermediate-low risk	393	77.6	0 (0)	346 (88.0)	47 (11.9)
Intermediate-high risk	57	11.2	0 (0)	25 (43.8)	32 (56.1)
High risk	14	2.7	0 (0)	1 (7.1)	13 (92.8)
Total	506	100	4 (0.7)	409 (80.3)	94 (18.3)

ESC: European Society of Cardiology. *The proportions refer to the total number of patients in each category. Note: Patients who were managed as recommended by the 2019 ESC guidelines are highlighted in gray.

A subgroup analysis was performed to evaluate adherence to recommendations on setting of care in cancer patients (Table 5). Of the total of patients with a history of cancer, 88.1% were classified as intermediate-low-risk patients, 9.4% were classified as intermediate-high-risk patients, and 2.3% were classified as high-risk patients, with no low-risk cases documented. None of the cancer patients were discharged early, the majority was managed in the general ward, and 50% of the intermediate-high-risk cases were monitored in the ICU. 

**Table 5:** Adherence to the European Society of Cardiology-recommended setting of care for cancer patients with acute pulmonary embolism of varying severity.

ESC classification	n	%	Setting of care		
			Early discharge, n (%)*	Hospitalization in the general ward, n (%)*	ICU, n (%)*
Low risk	0	0	0 (0)	0 (0)	0 (0)
Intermediate-low risk	149	88.1	0 (0)	137 (91.9)	12 (8.0)
Intermediate-high risk	16	9.4	0 (0)	8 (50.0)	8 (50.0)
High risk	4	2.3	0 (0)	0 (0)	4 (100)
Total	169	100	0 (0)	145 (85.8)	24 (14.2)

ESC: European Society of Cardiology. *The proportions refer to the total number of patients in each category.

Regarding anticoagulation therapy, all of the patients in the present study received initial anticoagulation with low-molecular-weight heparin. At the time of discharge, 203 (40.1%) of the patients were sent home on low-molecular-weight heparin, 34 (6.7%) were sent home on a vitamin K antagonist, and 213 (42.1%) were sent home on direct oral anticoagulants. Of the remaining 56 patients, 47 (9.3%) died in hospital and 9 (1.8%) were discharged without anticoagulation, because of bleeding complications. 

## DISCUSSION

The present study shows the rates of adherence to the 2019 ESC guideline recommendations on the setting of care for patients with acute PE of varying severity in a referral hospital in Colombia. Inadequate adherence was observed in the group of low-risk patients, most of whom were not discharged early as recommended. Similarly, in the intermediate-high-risk group, there was inadequate adherence to recommendations for monitoring in the ICU. 

The patients included in the present study had demographic characteristics and comorbidities that were similar to those reported in previous studies of populations diagnosed with PE,^12^ although the incidence of cancer was higher than the approximately 20% reported elsewhere.^13^ ^-^ ^15^ The distribution of cancer types in our study sample was similar to that in the general population, with gastrointestinal cancer being the most common.^16^ We used the sPESI because it is a widely validated tool for PE risk stratification, especially to identify low-risk patients.^17^ ^,^ ^18^ Regarding the frequency of risk groups, we found a lower proportion of low-risk patients.^17^ This might be due to a higher incidence of cancer in our sample, with most patients having an sPESI > 1. 

Our mortality rate was significantly higher than that reported in the literature. In the high-risk group, we found an in-hospital mortality of 35% in comparison with the 25% described in the general population; on the other hand, the in-hospital mortality in the intermediate-risk group was 8-14% in comparison with the 2-6% reported in previous studies.^19^ Thirty-day mortality was also higher in the present study: 42% in the high-risk group vs. 20% in previous studies; 22% in the intermediate-high-risk group vs. 8% in previous studies; and 18% in the intermediate-low-risk group vs. 6% in previous studies.^17^ These findings might be due to a higher incidence of cancer in our study population, especially given that more than half of the patients had a history of cancer at the time of death, with 94.2% having active cancer. 

In terms of adherence to recommendations for the setting of care for PE, it is noteworthy that only 9.5% of the patients classified as low-risk patients were discharged early, with a median hospital stay of 2 days, staying as long as 7 days in 25% of cases. Similar results have been observed in other studies, in which approximately 30-55% of low-risk patients are candidates for early discharge but in actual clinical practice 80-90% receive inpatient care.^5^ ^,^ ^6^ Outpatient treatment rates may vary across countries, being as low as 2% in Italy and 4% in France,^20^ both of which are even lower than those reported in the present study. These findings could be explained by differences across health care systems, as well as the perception of each physician regarding the management of low-risk PE and a possible lack of knowledge of the literature supporting the safety of early discharge in these cases.^21^ There are several risk prediction models such as the PESI, the sPESI, and the Hestia criteria to help clinicians stratify patients into appropriate risk groups. Nevertheless, an international survey of different European scientific societies found that the most commonly used prognostic tool for PE was clinical judgment, with prognostic models such as the PESI/sPESI used in only 30% of cases.^22^ In addition, other clinical and/or sociodemographic factors, such as social support network and access to health services, may have influenced the decision for early discharge. We do not have sufficient data on socioeconomic conditions, access to health care, or the possibility of post-discharge monitoring; therefore, we cannot be certain as to why inpatient management was decided on. The guidelines state that the decision for early discharge depends on social aspects such as access to anticoagulants, access to health care, and treatment monitoring after discharge. Thus, the decision for in-hospital management is correct in cases in which the conditions for early discharge are not met, a recommendation that also appears in the clinical practice guideline. 

In the present study, the recommendation to monitor intermediate-high-risk patients was found to be poorly adhered to, with approximately half of the cases being managed in the general ward rather than in the ICU as recommended.^23^ These results could also be explained by a lack of knowledge of the recommendations of the guidelines and the current evidence. Another explanation could be the presence of active cancer in most of the intermediate-high-risk patients admitted to the general ward. The prognosis of cancer may have been a contraindication to ICU admission or even the use of systemic thrombolysis. In addition, the availability of resources in the Colombian health care system, where access to an ICU is not immediate, may have been another reason why 40% of the patients in the present study did not receive close monitoring despite the indication. 

The results of the present study show that despite robust evidence for risk-adjusted management strategies for PE, including recommendations for setting of care, there is inadequate adherence to these recommendations, particularly for low-risk and intermediate-risk patients. This suggests that there are physician-related, clinical, sociodemographic, and health system-related factors that are barriers to adequate adherence to these recommendations. The objective of the present study was not to determine the factors influencing decisions regarding the setting of care, particularly outpatient management. Our results describe the setting of care in which patients with PE were treated, but further studies are needed to assess factors associated with this decision so that we can propose different strategies to promote adherence to guideline recommendations on the setting of care. 

The main limitation of the present study is its retrospective nature. However, missing data were minimal and did not significantly affect the results. Nevertheless, although the results of this study suggest a lack of adherence to recommendations regarding the setting of care for PE treatment, we did not identify the factors that influenced this decision or the reasons for not choosing the expected setting of care. We used an objective and validated prognostic score (the sPESI) in conjunction with other criteria in the risk model included in the 2019 ESC guidelines for the diagnosis and management of acute PE. However, there are other risk models to identify low-risk patients, such as the Hestia criteria, which include not only objective measures of risk but also other reasons for hospitalization, clinical situations that may require closer monitoring, and medical or social reasons for admission. We decided to use the sPESI because it is easier to retrieve data from electronic medical records, and studies have shown that the strategies based on the Hestia criteria and those based on the sPESI have similar safety and efficacy.^18^ Nevertheless, both tools need to be complemented by the judgment of the attending physician, given that in approximately 12% of patients eligible for outpatient management in accordance with the sPESI, the decision is overruled by the attending physician for other reasons. Future studies should focus on assessing the factors that influence the decision for outpatient or inpatient treatment, as well as the decision of whether or not to monitor patients in the ICU. 

In conclusion, the results of the present study suggest that clinical practice guideline recommendations regarding the setting of care for patients with acute PE are not being followed. This is particularly true for low-risk PE patients who may be candidates for early discharge. Further studies are needed to investigate the reasons why low-risk patients are not being discharged early. 
